# RepeatFiller newly identifies megabases of aligning repetitive sequences and improves annotations of conserved non-exonic elements

**DOI:** 10.1093/gigascience/giz132

**Published:** 2019-11-19

**Authors:** Ekaterina Osipova, Nikolai Hecker, Michael Hiller

**Affiliations:** 1 Max Planck Institute of Molecular Cell Biology and Genetics, Pfotenhauerstr. 108, 01307 Dresden, Germany; 2 Max Planck Institute for the Physics of Complex Systems, Noethnitzer Str. 38, 01187 Dresden, Germany; 3 Center for Systems Biology, Pfotenhauerstr. 108, 01307 Dresden, Germany

**Keywords:** transposons, conserved non-exonic elements, genome alignments

## Abstract

**Background:**

Transposons and other repetitive sequences make up a large part of complex genomes. Repetitive sequences can be co-opted into a variety of functions and thus provide a source for evolutionary novelty. However, comprehensively detecting ancestral repeats that align between species is difficult because considering all repeat-overlapping seeds in alignment methods that rely on the seed-and-extend heuristic results in prohibitively high runtimes.

**Results:**

Here, we show that ignoring repeat-overlapping alignment seeds when aligning entire genomes misses numerous alignments between repetitive elements. We present a tool, RepeatFiller, that improves genome alignments by incorporating previously undetected local alignments between repetitive sequences. By applying RepeatFiller to genome alignments between human and 20 other representative mammals, we uncover between 22 and 84 Mb of previously undetected alignments that mostly overlap transposable elements. We further show that the increased alignment coverage improves the annotation of conserved non-exonic elements, both by discovering numerous novel transposon-derived elements that evolve under constraint and by removing thousands of elements that are not under constraint in placental mammals.

**Conclusions:**

RepeatFiller contributes to comprehensively aligning repetitive genomic regions, which facilitates studying transposon co-option and genome evolution. Source code: https://github.com/hillerlab/GenomeAlignmentTools

## Introduction

A substantial portion of vertebrate genomes consist of transposons and other repetitive sequences [1, 2]. While most repeats are estimated to evolve neutrally [3], transposons are important substrates for evolutionary tinkering [4, 5]. For example, transposon-derived sequences contribute to the transcriptome by providing alternatively spliced exons [6, 7]. By contributing transcription factor binding sites, promoters, and distal regulatory elements, co-opted transposons are involved in rewiring of regulatory networks and drive regulatory innovation [7–15]. Importantly, a sizeable portion of evolutionarily constrained regions arose from ancestral transposon sequences [16, 17]. Studying how ancestral transposons and other repeats were co-opted into functional roles requires whole-genome alignments that comprehensively align orthologous repeats.

The nature of repetitive sequences such as transposons, however, leads to many paralogous alignments, which pose a challenge for comprehensively aligning orthologous repeats between vertebrate genomes. Most methods for aligning entire genomes use a seed-and-extend heuristic, originally implemented in BLAST [18], to find local alignments between the sequences of 2 genomes. The seeding step of this heuristic detects short words or patterns (called seeds) that match between the sequences of the 2 genomes. This can be computed very efficiently. Seed detection is then followed by a computationally more expensive alignment extension step that considers ungapped and gapped local alignments. Given that repetitive sequences provide numerous seed matches to paralogous repeat copies in a whole-genome comparison, it is computationally infeasible to start a local alignment from seeds located in repetitive sequences. Therefore, seeds that overlap repetitive regions are not used to start a local alignment phase, either by masking repetitive regions before aligning genomes [19–22] or by dynamically adapting seeding parameters by the observed seed frequencies [23]. Consequently, alignments between repeats are only found during the extension phase, initiated from seeds outside the repeat boundaries. This can be problematic if the regions flanking a repeat have been diverged to an extent that no seed in the vicinity of the repeat can be found.

Here, we investigated to what extent aligning repetitive sequences are missed in whole-genome alignments. We show that ignoring repeat-overlapping seeds misses between 22 and 84 Mb of mostly repetitive elements that actually align between mammals and we provide a tool, called RepeatFiller, to incorporate such repeat-overlapping alignments into genome alignments. We further show that a subset of aligning sequences detected by RepeatFiller evolve under evolutionary constraint, which uncovers previously unknown conserved non-exonic elements (CNEs) and thus improves the annotation of constrained elements.

## Results

### RepeatFiller incorporates several megabases of aligning repetitive sequences to mammalian genome alignments

**Figure 1:**
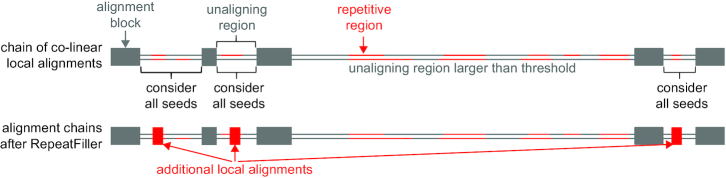
Missed repeat-overlapping alignments and concept of RepeatFiller. Illustration of RepeatFiller. Focusing on unaligning regions in a reference and query genome that are flanked by up- and downstream aligning blocks, RepeatFiller performs a second round of local alignment considering also repeat-overlapping seeds. Newly found local alignments (red boxes) are inserted into the context of other aligning blocks (grey boxes). Unaligning regions that are larger than a user-defined threshold are not considered because the chance of aligning non-orthologous repeats is increased.

To investigate how many aligning repetitive elements have been missed in alignments between mammalian genomes, we adopted a previously developed approach that was initially devised to detect novel local alignments between a pair of distantly related species [24, 25]. The original approach focused on unaligning regions that are flanked by aligning blocks in co-linear alignment chains [26], which are detected in the first all-vs-all genome alignment step. In a second step, this original approach used LASTZ [21] with highly sensitive seeding and (un)gapped extension parameters to align the previously unaligning regions again. This second round of highly sensitive local alignment can uncover novel alignments that are co-linear with already-detected alignment blocks. Here, we adopted this approach by introducing 2 key changes. First, we increased alignment parameter sensitivity only slightly but unmasked the unaligning region. This implies that all seeds, including repeat-overlapping seeds, will be considered (Fig. 1). By restricting the size of the unaligning regions to smaller regions of ≤20 kb, we reason that novel local alignments detected with a similar sensitivity level likely constitute orthologous alignments. Second, while the previous approach computed all alignment chains again from scratch using previously detected and novel local alignments, our new approach directly adds novel alignments to existing alignment chains, thus removing the need for a chain recomputing step. This approach is called RepeatFiller [27].

**Figure 2:**
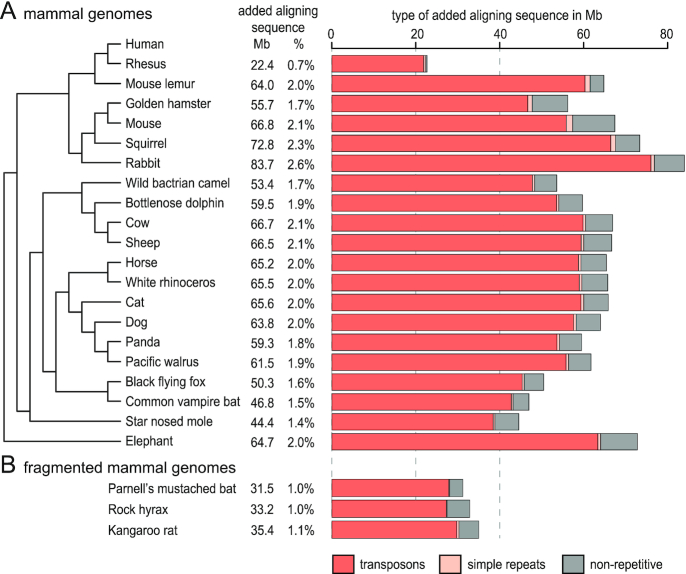
RepeatFiller adds several megabases of aligning transposable elements to existing mammalian genome alignments. (A) Phylogenetic tree of human and 20 non-human mammals whose genomes we aligned to the human genome. The amount of new alignments detected by RepeatFiller is shown in megabases and in percent relative to the human genome. Bar charts provide a breakdown of newly added aligning sequences into overlap with transposons, simple repeats, and non-repetitive sequence. (B) Application of RepeatFiller to fragmented mammalian assemblies still adds a substantial amount of new alignments.

To investigate how many aligning repetitive elements can be added by RepeatFiller, we built alignment chains between the human (hg38) genome assembly and the genomes of 20 other mammals that represent the major mammalian clades (Fig. 2A, Supplementary Table 1). We found that RepeatFiller adds between 22.4 Mb (rhesus macaque) and 83.7 Mb (rabbit) of aligning sequence, which represents 0.7–2.6% of the human genome (Fig. 2A, Supplementary Table 1). RepeatFiller added fewer new alignments for the rhesus macaque likely because the genomes of both species are very similar (their evolutionary distance is <0.07 substitutions per neutral site). This makes it more likely to find seeds outside of masked repetitive regions and to extend alignments into repeats during the extension phase. By overlapping the new alignments with repetitive elements annotated in the human genome, we found that the vast majority of newly aligned sequences overlap repeats, in particular transposable elements (Fig. 2A, Supplementary Table 1). The runtime of the RepeatFiller step is between 14.7 and 43.4 CPU hours (Supplementary Table 1) and thus adds little to the runtime of the initial genome-wide all-vs-all pairwise alignment step, which is typically ∼1,000 CPU hours.

Next, we investigated what factors are associated with differences in the amount of newly aligned sequences per species. In these tests, we excluded rhesus macaque, which is closely related to human, as an outlier. First, as expected, the percent of the genome that is repeat-masked significantly influences the number of newly aligned bases (*P* = 0.0004, Supplementary Table 1), which supports our assumption that the initial alignment step misses alignments owing to repeat-masking rather than sequence dissimilarity. Second, we investigated how assembly contiguity of the query genome influences the results. We found that the scaffold N50 value has a small but non-significant effect on the amount of added aligned bases (*P* = 0.067, Supplementary Table 1). Because chains cannot span scaffold boundaries, we further tested the influence of scaffold N50 values by applying RepeatFiller to alignments of 3 fragmented mammalian assemblies: Parnell's mustached bat, rock hyrax, and kangaroo rat, which have scaffold N50 values between 23 and 36 kb. While RepeatFiller still added a substantial amount of new alignments, ranging from 32 to 35 Mb (Fig. 2B, Supplementary Table 2), more new alignments were generally found for more contiguous mammalian assemblies. Together, this shows that a considerable portion of aligning transposon sequences are missed when repeat-overlapping seeds are ignored and that for both fragmented and contiguous mammalian genomes RepeatFiller can detect such alignments with little extra computational runtime.

### RepeatFiller also detects additional alignments for non-mammalian genomes

**Figure 3:**
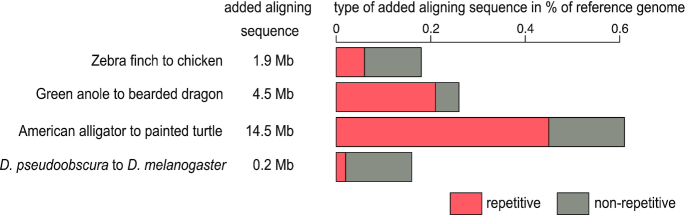
RepeatFiller also detects additional alignments for non-mammalian genomes. The figure shows how many new alignments were detected by applying RepeatFiller to pairwise alignments of birds, reptiles, and drosophilids. Both the amount (in megabases) of new alignments and the percent of the reference genome additionally aligned are shown. Bar charts show which portion of newly added alignments overlap repetitive sequences.

The majority of the newly detected alignments between mammalian genomes overlap transposable elements or other repeats. One would therefore expect RepeatFiller application to alignments of species with less repeat-rich genomes to detect fewer novel alignments. To test whether this is generally true, we applied RepeatFiller to alignments of birds (zebra finch aligned to chicken), reptiles (green anole aligned to bearded dragon; American alligator aligned to painted turtle), and insects (*Drosophila pseudoobscura* aligned to *Drosophila melanogaster*). For birds and insects, whose genomes generally consist of <20% repeats [28–30], RepeatFiller added few new alignments (1.9 Mb for birds, representing 0.18% of the chicken genome; 231 kb for drosophilids, representing 0.16% of the *D. melanogaster* genome) (Fig. 3, Supplementary Table 2). For reptiles, RepeatFiller added 4.5 Mb of new alignments to the green anole–bearded dragon genome alignment (0.26% of the bearded dragon genome) and 14.5 Mb to the alligator–turtle alignment (0.61% of the turtle genome) (Fig. 3, Supplementary Table 2). Thus, despite the fact that reptile and mammal genomes generally have a similar repeat content of ∼30–50% [29, 31], RepeatFiller added fewer alignment for reptiles than for mammals. This shows that other factors in addition to genomic repeat content also influence the amount of added alignments. Nevertheless, >1 Mb of previously undetected alignments for birds or reptiles shows that RepeatFiller, with little additional runtime, can also improve the completeness of aligning repetitive regions between species in these groups.

### RepeatFiller application uncovers thousands of novel repeat-derived conserved non-exonic elements

Next, we investigated whether some of the newly aligning sequences show evidence of evolutionary constraint, which indicates purifying selection and a biological function. To this end, we used the pairwise alignments, generated either with or without RepeatFiller, to build 2 human-referenced multiple genome alignments of 21 mammals with Multiz [32]. Then, we used PhastCons [33] to identify constrained elements. We found that the majority (98%) of the 164 Mb in the human genome that are classified as constrained in the multiple alignment without RepeatFiller were also classified as constrained in the RepeatFiller-subjected alignment.

Dividing the conserved regions detected in the alignment without RepeatFiller into exonic and non-exonic regions, we found that 99.8% of the exonic and 97.4% of the non-exonic regions are also classified as constrained in the RepeatFiller-subjected alignment. Because conserved exonic regions are virtually identical, likely because they rarely overlap repeats, we focused our comparison on the conserved non-exonic elements (CNEs), which often overlap *cis*-regulatory elements [34–36]. This comparison first showed that 3.46 Mb of the human genome were newly classified as conserved non-exonic in the RepeatFiller-subjected alignment, representing 2.9% of all conserved non-exonic bases detected in this alignment. Requiring a minimum size of 30 bp, application of RepeatFiller led to the identification of 30,167 novel CNEs that are listed in Supplementary Table 3. With a median size of 41 bp, these novel CNEs are shorter than CNEs already detected in the non-RepeatFiller alignments (median 50 bp, 2-sided Wilcoxon rank sum test *P* < E−16, Supplementary Fig. 1), likely because most of the longer conserved regions were already in the initial genome-wide alignment step. Consistent with previous findings that CNEs are in general more AT-rich [37], we found that the novel CNEs are more AT-rich than randomly selected, non-conserved genomic regions (2-sided Wilcoxon rank sum test *P* < E−16, Supplementary Fig. 1).

**Figure 4:**
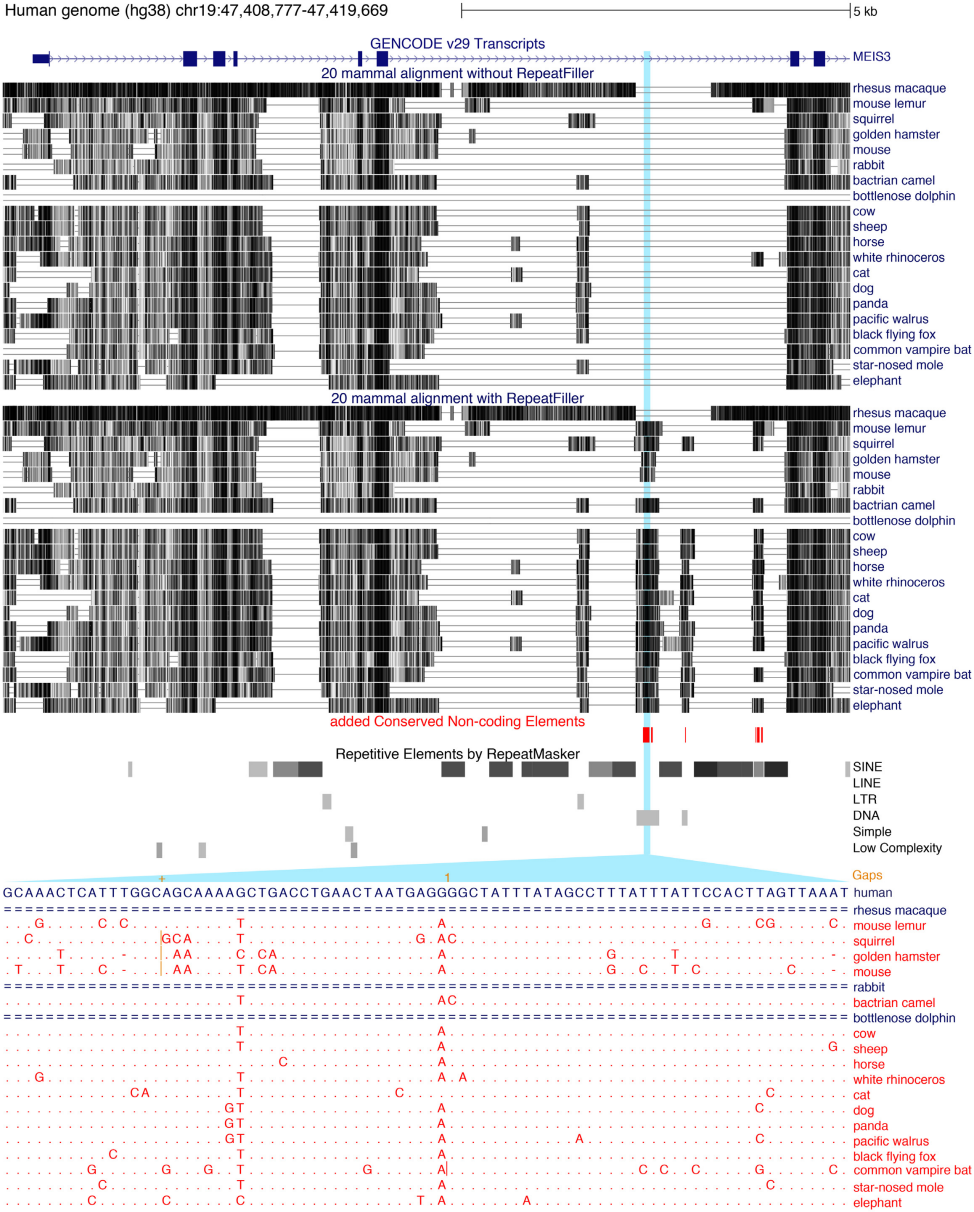
Examples of newly identified CNEs near *MEIS3*. UCSC genome browser [45] screenshot shows an ∼11 kb genomic region overlapping the gene *MEIS3*, a homeobox transcription factor that is required for hindbrain development. Visualization of the 2 multiple genome alignments (without RepeatFiller at the top, with RepeatFiller below; boxes representing align regions with darker colors indicating a higher alignment identity) shows that RepeatFiller adds several aligning sequences, some of which evolve under evolutionary constraint and thus are CNEs (red boxes) only detected in the RepeatFiller-subjected alignment. The RepeatMasker annotation shows that these newly identified CNEs overlap transposons. The zoom-in shows the 21-mammal alignment of one of the newly identified CNEs, which overlaps a DNA transposon. While this genomic region did not align to any mammal before applying RepeatFiller, our tool identified a well-aligning sequence for 17 non-human mammals (red font). A dot represents a base that is identical to the human base, insertions are marked by vertical orange lines, and unaligning regions are showed as double lines.

**Figure 5:**
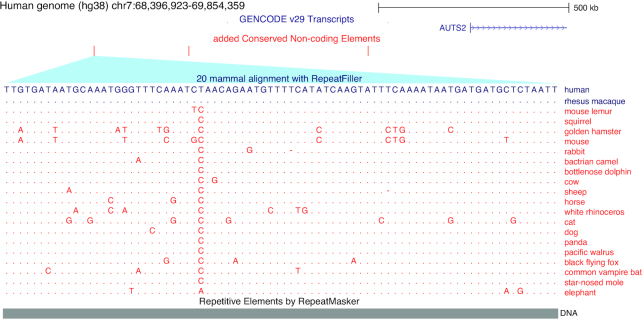
Examples of newly identified CNEs upstream of *AUTS2*. UCSC genome browser screenshot shows a ∼1.5 Mb genomic region around *AUTS2*, a transcriptional regulator required for neurodevelopment. CNEs only detected in the RepeatFiller-subjected multiple alignment are marked as red tick marks. The zoom-in shows the 21-mammal alignment of one of the newly identified CNEs. While only the rhesus macaque sequence aligned to human before applying RepeatFiller, our tool identifies a well-aligning sequence for all 19 other mammals (red font). A dot represents a base that is identical to the human base. The RepeatMasker annotation (bottom) shows that this newly identified CNE overlaps a DNA transposon.

Two striking examples of newly identified CNEs are shown in Figs 4 and 5. Fig. 4 shows the genomic region overlapping *MEIS3*, a homeobox transcription factor gene that synergizes with Hox genes and is required for hindbrain development and survival of pancreatic β-cells [38–40]. By revealing novel alignments to many non-human mammals, RepeatFiller identifies several novel repeat-overlapping CNEs in introns of *MEIS3* (Fig. 4). Fig. 5 shows the genomic region around *AUTS2*, a transcriptional regulator required for neurodevelopment that is associated with human neurological disorders such as autism [41, 42]. Applying RepeatFiller revealed several novel CNEs upstream of *AUTS2*. For some of these CNEs, RepeatFiller incorporated a well-aligning sequence of 19 mammals, which then permitted the identification of evolutionary constraint. Overall, applying RepeatFiller led the identification of >30,000 CNEs that were not detected before.

### RepeatFiller improves annotations of Conserved Non-exonic Elements

**Figure 6:**
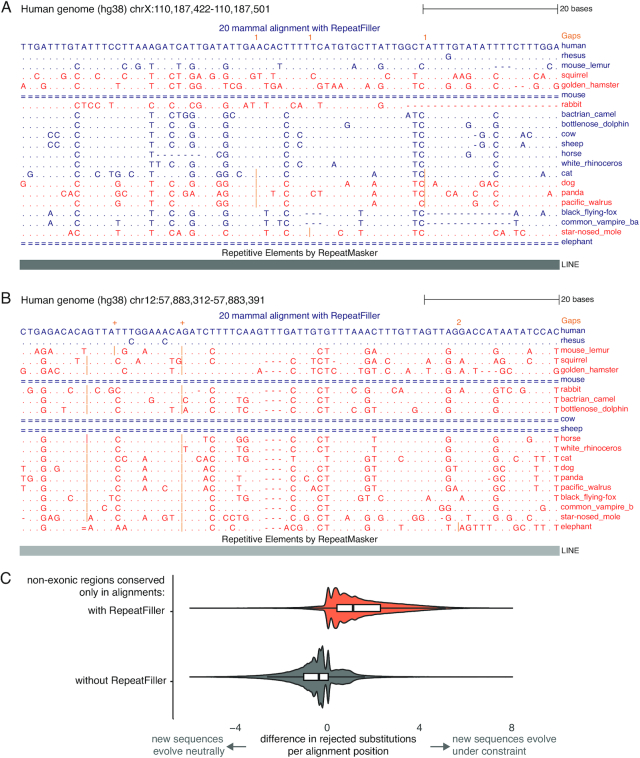
Additional alignments found with RepeatFiller reveal absence of conservation in the genomic regions that were erroneously classified as conserved before. (A, B) UCSC genome browser screenshots showing 2 examples of genomic regions that were only classified as constrained in a multiple genome alignment generated without applying RepeatFiller. Dots in these alignments represent bases that are identical to the human base, insertions are marked by vertical orange lines, and unaligning regions are shown as double lines. The alignments show that the sequences of species added by RepeatFiller (red font) exhibit a number of substitutions. This explains why these regions were not classified as constrained anymore, despite adding more aligning sequences. Note that in (B) only the sequence of the rhesus macaque was aligned before applying RepeatFiller. Sequences in both (A) and (B) overlap long interspersed nuclear element transposons (LINEs). (C) Difference in evolutionary constraint in non-exonic alignment columns that are only classified as constrained in either alignment. For each alignment position, we used GERP++ to compute the estimated number of substitutions rejected by purifying selection (RS). The difference in RS between alignments with and without RepeatFiller is visualized as a violin plot overlaid with a white box plot (box spans the first to third quartile and indicates the median). This shows that almost all non-exonic bases that were only detected as constrained in the alignment with RepeatFiller (orange background) have a positive RS difference, indicating that the newly aligning sequences added by RepeatFiller largely evolve under evolutionary constraint. In contrast, non-exonic bases only detected as constrained in the alignment without RepeatFiller (grey background) often have slightly negative RS differences, indicating that many of the newly added sequences do not evolve under constraint. The 2 distributions are significantly different (*P* < E−16, 2-sided Wilcoxon rank sum test).

Interestingly, the comparison of conserved non-exonic bases detected by PhastCons also revealed 3.08 Mb of the human genome that were classified as conserved non-exonic only in the multiple alignment without RepeatFiller, but not in the RepeatFiller-subjected alignment. These 3.08 Mb represent 2.6% of all conserved non-exonic bases detected in the alignment without RepeatFiller. The 29,334 CNEs with a size ≥30 bp are listed in Supplementary Table 4. To investigate the reasons underlying these “lost” CNEs, we first sought to confirm that the RepeatFiller-subjected alignment had an increased species coverage in these regions. Indeed, we found that RepeatFiller added on average 3.9 (median 3) aligning species to these lost CNEs. Inspecting many of these CNEs showed that the newly added sequences are similar to the already-aligned sequences; however, they exhibit more substitutions. These substitutions increase the overall sequence divergence across mammals, which likely explains why the same region was not classified as constrained anymore, despite having a higher coverage of aligning species. Fig. 6A and B shows 2 examples of such genomic regions that are not classified as constrained after adding additional alignments with RepeatFiller.

To confirm that the newly added sequences increase the overall sequence divergence, we applied GERP++ [43] to both multiple alignments (Supplementary Fig. 2A). For each alignment column, GERP++ estimates the number of substitutions that were rejected by purifying selection (RS = rejected substitutions) by subtracting the number of observed substitutions from the number of substitutions expected under neutrality. Because GERP++ computes the number of substitutions expected under neutrality from a phylogenetic tree that is pruned to the aligning species (Supplementary Fig. 2B), we can directly compare RS between alignment columns that were only classified as constrained in either alignment to estimate whether the RepeatFiller-added sequences evolve slower than expected under neutrality. Specifically, for each alignment column, we computed the difference in RS before and after adding new alignments with RepeatFiller, as illustrated in Supplementary Fig. 2B.

We found that the alignment columns, where constraint was only detected in the alignment without RepeatFiller, mostly exhibit slightly negative RS differences (Fig. 6C, grey background), which suggests that many positions in the RepeatFiller-added sequences do not evolve under strong constraint. Hence, the extent of constraint in the more limited set of aligning sequences was likely overestimated, providing an explanation of why these genomic regions were not classified anymore as constrained across placental mammals. It should be noted that these regions may still be under constraint in particular lineages. In contrast, most alignment columns, where constraint was only detected after applying RepeatFiller, exhibit a positive RS difference (Fig. 6C, orange background), which suggests that the newly added sequences evolve under constraint. Overall, by uncovering previously unknown alignments, RepeatFiller application led to an improved CNE annotation.

## Discussion

While transposon-derived sequences can be co-opted into a multitude of biological roles and can evolve under evolutionary constraint, comprehensively detecting alignments between ancestral transposons and other repeats is not straightforward. The main reason is that considering all repeat-overlapping alignment seeds during the initial whole-genome alignment step is computationally not feasible. However, it is feasible to consider all seeds when aligning local regions that are bounded by colinear aligning blocks. We provide a tool, RepeatFiller, that implements this idea and incorporates newly detected repeat-overlapping alignments into pairwise alignment chains. We tested the tool on alignments between human and 20 representative mammals and showed that with little additional computational runtime RepeatFiller uncovers between 22 and 84 Mb of previously undetected alignments that mostly originate from transposable elements. We also showed that RepeatFiller can detect megabases of previously undetected alignments for fragmented mammalian genomes or for genomes of birds and reptiles, suggesting that RepeatFiller can be applied to genome alignments of a wide range of species.

We further show that RepeatFiller application enables a refined and more complete CNE annotation by 2 means. First, applying RepeatFiller led to the identification of thousands of CNEs whose aligning sequences were not detected before. This includes highly conserved transposon-derived CNEs that are located near important developmental genes. Second, the sequences added by RepeatFiller may not evolve slower than expected under neutral evolution. In this case, providing a more complete set of aligning sequences led to the removal of thousands of putatively spurious CNEs that overall do not evolve under strong constraint across placental mammals, although the possibility of lineage-specific constraint remains.

Taken together, RepeatFiller implements an efficient way to improve the completeness of aligning repetitive regions in whole-genome alignments, which helps in annotating CNEs and studying transposon co-option and genome evolution.

## Materials and Methods

### Generating pairwise genome alignments

For all mammalian species, we used the human hg38 genome assembly as the reference genome. For the alignments of non-mammalian species, the reference assemblies are specified in Supplementary Table 2. To compute pairwise genome alignments, we used LASTZ version 1.04.00 [21] and the chain/net pipeline [26] with default parameters (chainMinScore 1000, chainLinearGap loose). We used the LASTZ alignment parameters K = 2,400, L = 3,000, Y = 9,400, H = 2,000, and the LASTZ default scoring matrix. We also tested aligning human and rhesus macaque using K = 4,500, L = 3,000, Y = 15,000, H = 2,000, and the UCSC human_chimp.v2.q scoring matrix and found that applying RepeatFiller to these chains also added a similar amount (25.2 vs 22.4 Mb) of newly aligning sequence (Supplementary Table 1). All species names and their assemblies are listed in Supplementary Tables 1 and 2.

### RepeatFiller

The input of RepeatFiller is a file containing co-linear chains of local alignment blocks. This file must be in the UCSC chain format as defined here [44]. The output is a file that contains the same chains plus the newly added local alignment blocks. By default, RepeatFiller only considers unaligned regions in both the reference and query genome that are ≥30 and ≤20,000 bp long. We considered all chains with the score >25,000. For each unaligning region that fulfills the size thresholds, RepeatFiller uses LASTZ with the same parameters as above but with a slightly more sensitive ungapped alignment threshold (K = 2,000). All repeat-masking (lowercase letters) was removed before providing the local sequences to LASTZ. Because LASTZ may find multiple additional local alignments in this second step, we used axtChain [26] to obtain a “mini chain” of local alignments for this unaligning region. RepeatFiller then inserts the aligning blocks of a newly detected mini chain at the respective position in the original chain if the score of the mini chain is ≥5,000. All default parameters for the size of unaligning regions, minimum chain scores, and local alignment parameters can be changed by the user via parameters. Finally, RepeatFiller recomputes the score of the entire chain if new alignments were added.

We compared the number of aligning bases in the chains before and after applying RepeatFiller. To this end, we used the coordinates of aligning chain blocks to determine how many bases of the human hg38 assembly align (via ≥1 chain) to the query species. We used the RepeatMasker repeat annotation for hg38, available at the UCSC Genome Browser [45], to determine how many of the newly added alignments overlap repetitive elements.

### Generating multiple alignments

Before building multiple alignment, we filtered out low-scoring chains and nets, requiring a minimum score of 100,000. We used Multiz-tba [32] with default parameters to generate 2 reference-based multiple alignments using the pairwise alignment nets produced with and without RepeatFiller, respectively.

### Conservation analysis

To identify constrained elements, one needs a tree with branch lengths representing the number of substitutions per neutral site. We used 4-fold degenerated codon sites based on the human Ensembl gene annotation to estimate the neutral branch lengths with PhyloFit [33]. To identify conserved regions, we used PhastCons [33] with the following parameters: rho = 0.31; expected-length = 45; target-coverage = 0.3. To obtain conserved non-exonic regions, we first obtained exonic regions from the human Ensembl and RefSeq annotation (UCSC tables ensGene and refGene). As done before [25], we merged all exonic regions and added 50 bp flanks to exclude splice site proximal regions that often harbor conserved splicing regulatory elements. To obtain CNEs, we subtracted these exonic bases and their flanks from all conserved regions.

To compare constraint in genomic regions classified as constraint in only 1 alignment, we used GERP++ (RRID:SCR_000563) [43] with default parameters (acceptable false-positive rate = 0.05) to estimate constraint per genomic position. We denote genomic regions as “gained” if they were classified as constrained by PhastCons only in the multiple alignment generated with RepeatFiller. We denote genomic regions as “lost” if they were classified as constrained only in alignment generated without RepeatFiller (Supplementary Fig. 2A). Gained and lost regions were identified using “bedtools intersect” (RRID:SCR_006646) [46]. For each position in “gained” and “lost” non-exonic regions, we computed the RS score (number of rejected substitutions) with GERP++ [43] and calculated the difference between the RS score obtained for the alignment with and without RepeatFiller (Supplementary Fig. 2B). These differences are plotted in Fig. 6C. Positive differences indicate that the sequences added by RepeatFiller evolve slower than under neutrality, thus increasing the number of rejected substitutions. Differences close to zero indicate that the newly added sequences evolve as expected under neutral evolution, and negative differences indicate that they evolve faster than expected under neutral evolution.

## Availability of Supporting Source Code and Requirements

- Project name: RepeatFiller
- Project home page: https://github.com/hillerlab/GenomeAlignmentTools
- Programming languages: Perl and Python
- Other requirements: LASTZ
- License: MIT License
- RRID:SCR_017414
- ELIXIR bio.tools registry: biotools: RepeatFiller

## Availability of Supporting Data and Materials

The multiple genome alignments generated with and without applying RepeatFiller and the respective PhastCons conserved elements are available at https://bds.mpi-cbg.de/hillerlab/RepeatFiller/. The CNEs that differ between both alignments are available in Supplementary Tables 3 and 4. The RepeatFiller source code is available at https://github.com/hillerlab/GenomeAlignmentTools. Other supporting data and code snapshots are available from the *GigaScience* GigaDB repository [47].

## Additional files

Supplementary Figure 1: Properties of newly detected CNEs. We compared CNEs already detected in the non-RepeatFiller alignments (dark grey) to 30,167 novel CNEs that were only detected after applying RepeatFiller (orange). (A) Violin plots overlaid by box plots show that newly detected CNEs are significantly shorter (median 41 vs 50 bp; mean 50.3 vs 76.7 bp) and lack very large CNEs (maximum 760 vs 2,193 bp). For visualization, the shown distributions are cut at 200 bp. (B) The size distribution of newly and already-detected CNEs is similar to a power law distribution. (C) Violin plots overlaid by box plots show the percent A+T bases per CNE. Newly and already-detected CNEs are more AT-rich than randomly selected, non-conserved genomic regions of the same size as the already-detected CNEs. We repeated the sampling of non-conserved genomic regions 10 times and found a highly significant difference in each case.

***: *P* < E−16 in a 2-sided Wilcoxon rank sum test.

Supplementary Figure 2: Comparing constraint in conserved non-exonic regions that were only classified as conserved in the alignment with or without RepeatFiller. (A) Conserved non-exonic elements (CNEs) obtained by PhastCons for alignments with and without RepeatFiller (represented by light grey boxes) are largely identical. However, some of the regions are annotated as conserved either only in the RepeatFiller-subjected alignment (‘gained’ regions—orange boxes) or only in the alignment without RepeatFiller (‘lost’ regions—dark grey boxes). (B) For each position in these variable CNEs, we calculate the number of rejected substitutions (RS) with GERP++, separately for the alignments with and without RepeatFiller. The illustration shows that RepeatFiller adds more aligning sequences (red font). GERP++ computes the number of substitutions expected under neutrality from a phylogenetic tree that is pruned to the aligning species. That means that branches leading to non-aligning species (dashed grey lines) are ignored when computing the number of expected neutral substitutions. The difference in rejected substitutions per alignment column (plotted in Fig. 6C) is calculated as the difference of the 2 RS scores.

Supplementary Table 1: Species, assemblies, RepeatFiller statistics, and factors that might correlate with the amount of added alignments.

Supplementary Table 2: Testing RepeatFiller on species with fragmented assemblies and non-mammalian species.

Supplementary Table 3: CNEs that are only detected in a multiple genome alignment with RepeatFiller.

Supplementary Table 4: CNEs that are only detected in a multiple genome alignment without RepeatFiller.

giz132_GIGA-D-19-00249_Original_Submission

giz132_GIGA-D-19-00249_Revision_1

giz132_Response_to_Reviewer_Comments_Original_Submission

giz132_Reviewer_1_Report_Original_Submission R Daniel Kortschak -- 8/14/2019 Reviewed

giz132_Reviewer_2_Report_Original_Submission Ge Tan, Ph.D. -- 8/20/2019 Reviewed

giz132_Reviewer_2_Report_Revision_1 Ge Tan, Ph.D. -- 9/16/2019 Reviewed

giz132_Supplemental_Files

## Abbreviations

BLAST: Basic Local Alignment Search Tool; bp: base pairs; CNE: conserved non-exonic element; CPU: central processing unit; GERP: Genomic Evolutionary Rate Profiling; kb: kilobase pairs; Mb: megabase pairs; RS: rejected substitutions; UCSC: University of California Santa Cruz.

## Competing interests

The authors declare that they have no competing interests.

## Funding

This work was supported by the Max Planck Society and the Leibniz Association (SAW-2016-SGN-2).
