# Supplementary material for: RepeatFiller newly identifies megabases of aligning repetitive sequences and improves annotations of conserved non-exonic elements
Source: Gigascience. 2019 Nov 19;8(11):giz132. doi: 10.1093/gigascience/giz132 (PMC6862929; doi:10.1093/gigascience/giz132)
Supplement: giz132_Supplemental_Files [file giz132_supplemental_files.zip › Supplement.pdf]

Supplementary Information for

RepeatFiller newly identifies megabases of aligning repetitive sequences and improves annotations of conserved non-exonic elements

Ekaterina Osipova [1,2,3], Nikolai Hecker [1,2,3], Michael Hiller [1,2,3*]

[1]Max Planck Institute of Molecular Cell Biology and Genetics, Dresden, Germany
[2]Max Planck Institute for the Physics of Complex Systems, Dresden, Germany
[3]Center for Systems Biology Dresden, Germany

*To whom correspondence should be addressed:
Michael Hiller
Computational Biology and Evolutionary Genomics, Max Planck Institute of Molecular Cell Biology and Genetics & Max Planck Institute for the Physics of Complex Systems, Dresden, Germany.
Tel: +49 351 210 2781
Fax: +49 351 210 1209
Email: hiller@mpi-cbg.de

This PDF file contains:

- Supplementary Figures 1 - 2

Supplementary Tables 1 – 4 are provided as sheets in a separate Excel file.

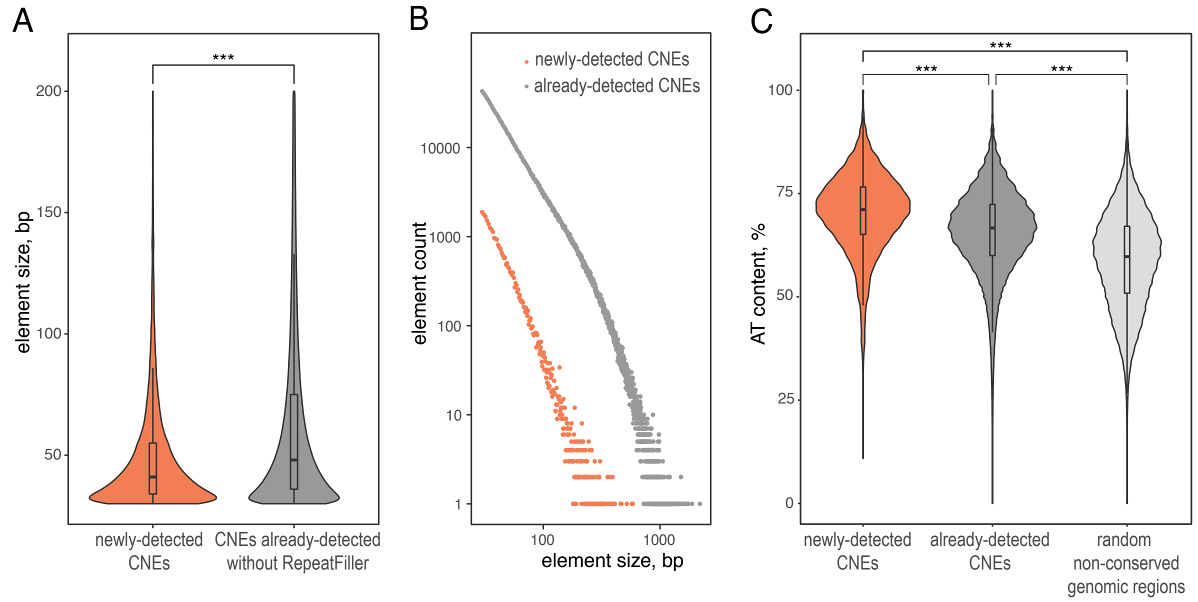

Supplementary Figure 1: Properties of newly-detected CNEs.

We compared CNEs already-detected in the non-RepeatFiller alignments (dark grey) to 30167 novel CNEs that were only detected after applying RepeatFiller (orange).

(A) Violin plots overlaid by box plots show that newly-detected CNEs are significantly shorter (median 41 vs. 50 bp, average 50.3 vs. 76.7 bp) and lack very large CNEs (maximum 760 vs. 2193 bp). For visualization, the shown distributions are cut at 200 bp.

(B) The size distribution of newly- and already-detected CNEs is similar to a power law distribution.

(C) Violin plots overlaid by box plots show the percent A+T bases per CNE. Newly- and already-detected CNEs are more AT-rich than randomly selected, non-conserved genomic regions that have the same size as the already-detected CNEs. We repeated the sampling of non-conserved genomic regions 10 times and found a highly significant difference in each case.

***: $P<e^{-16}$ in a two-sided Wilcoxon rank sum test.

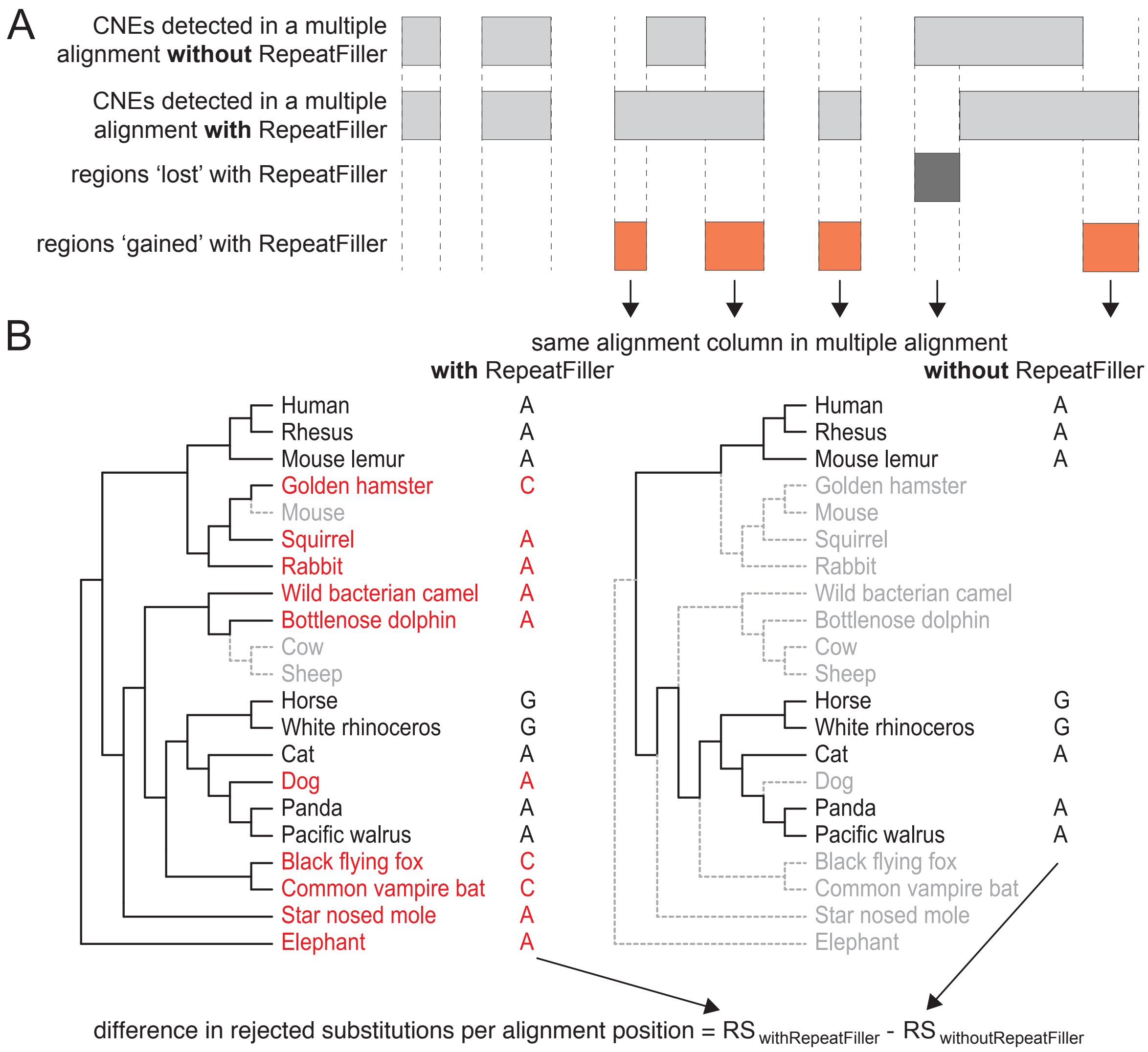

Supplementary Figure 2: Comparing constraint in conserved non-exonic regions that were only classified as conserved in the alignment with or without RepeatFiller.
(A) Conserved Non-exonic Elements (CNEs) obtained by PhastCons for alignments with and without RepeatFiller (represented by light grey boxes) are largely identical. However, some of the regions are annotated as conserved either only in the RepeatFiller-subjected alignment ('gained' regions – orange boxes) or only in the alignment without RepeatFiller ('lost' regions – dark grey boxes).
(B) For each position in these variable CNEs, we calculate the number of rejected substitutions (RS) with GERP++, separately for the alignments with and without RepeatFiller. The illustration shows that RepeatFiller adds more aligning sequences (red font). GERP++ computes the number of substitutions expected under neutrality from a phylogenetic tree that is pruned to the aligning species. That means that branches leading to non-aligning species (dashed grey lines) are ignored when computing the number of expected neutral substitutions. The difference in rejected substitutions per alignment column (plotted in Figure 6C) is calculated as the difference of the two RS scores.
