# Supplementary material for: RepeatFiller newly identifies megabases of aligning repetitive sequences and improves annotations of conserved non-exonic elements
Source: Gigascience. 2019 Nov 19;8(11):giz132. doi: 10.1093/gigascience/giz132 (PMC6862929; doi:10.1093/gigascience/giz132)
Supplement: giz132_Response_to_Reviewer_Comments_Original_Submission [file giz132_response_to_reviewer_comments_original_submission.pdf]

Author's Response To Reviewer Comments

Close

We have uploaded the point-by-point response as a separate pdf document in the category supplementary Data, but labeled as 'PointByPointResponse'.

Close
