# Supplementary material for: RepeatFiller newly identifies megabases of aligning repetitive sequences and improves annotations of conserved non-exonic elements
Source: Gigascience. 2019 Nov 19;8(11):giz132. doi: 10.1093/gigascience/giz132 (PMC6862929; doi:10.1093/gigascience/giz132)
Supplement: giz132_Reviewer_1_Report_Original_Submission — R Daniel Kortschak -- 8/14/2019 Reviewed [file giz132_reviewer_1_report_original_submission.pdf]

Reviewer Report

Title: RepeatFiller newly identifies megabases of aligning repetitive sequences and improves annotations of conserved non-exonic elements

Version: Original Submission **Date:** 8/14/2019

Reviewer name: R Daniel Kortschak

Reviewer Comments to Author:

"RepeatFiller newly identifies megabases of aligning repetitive sequences and improves annotations of conserved non-exonic elements" is a nice demonstration of a general software engineering approach to addressing high complexity problems, it reduces the complexity by making sensible cuts in the input data to make the problem feasible. In the case of RepeatFiller, sensible cuts are placed at the boundaries of otherwise uniquely unalignable segments of genomes based on anchor regions that are uniquely unalignable. This approach reduces the seed complexity of alignment from genome scale down to a scale that can be properly considered by standard aligners.
The results of using this approach show that known interspecies-conserved regions are both missed without the use of this technique due to aligners ignoring high-repeat density regions for alignment seeding, but also conversely that known conserved regions have their level of conversation overestimated due to absence of more divergent alignments when using traditional alignment methods. Given the apparent importance of repetitive elements in the evolution of eukaryotic gene regulation the tool described here is likely to find interesting new regulation systems and refine understanding of existing systems.
The manuscript is well written with only a minor grammatical error: "Requiring minimum size of 30 bp, application [of] RepeatFiller led to the identification of ...".
It would be helpful for the authors to provide better instructions for building the dependencies (downstream dependencies of Kent's source are not listed either on the project page or in the build instructions for the Kent source). It should be noted that attempting to build Kent's source fails for absence of dependencies that appear not to be used by RepeatFiller (MySQL). The provided pre-compiled binaries do not run, again due to a missing (or mis-versioned) dependency (libssl). Because of these issues it may be worth vendoring a cut down Kent source with the RepeatFiller source.

Level of Interest

Please indicate how interesting you found the manuscript: Choose an item.

Quality of Written English

Please indicate the quality of language in the manuscript: Choose an item.

Declaration of Competing Interests

Please complete a declaration of competing interests, considering the following questions:

- Have you in the past five years received reimbursements, fees, funding, or salary from an organisation that may in any way gain or lose financially from the publication of this manuscript, either now or in the future?
- Do you hold any stocks or shares in an organisation that may in any way gain or lose financially from the publication of this manuscript, either now or in the future?
- Do you hold or are you currently applying for any patents relating to the content of the manuscript?
- Have you received reimbursements, fees, funding, or salary from an organization that holds or has applied for patents relating to the content of the manuscript?
- Do you have any other financial competing interests?
- Do you have any non-financial competing interests in relation to this paper?

If you can answer no to all of the above, write 'I declare that I have no competing interests' below. If your reply is yes to any, please give details below.

I declare that I have no competing interests.

I agree to the open peer review policy of the journal. I understand that my name will be included on my report to the authors and, if the manuscript is accepted for publication, my named report including any attachments I upload will be posted on the website along with the authors' responses. I agree for my report to be made available under an Open Access Creative Commons CC-BY license (http://creativecommons.org/licenses/by/4.0/). I understand that any comments which I do not wish to be included in my named report can be included as confidential comments to the editors, which will not be published.

Choose an item.

To further support our reviewers, we have joined with Publons, where you can gain additional credit to further highlight your hard work (see: https://publons.com/journal/530/gigascience). On publication of this paper, your review will be automatically added to Publons, you can then choose whether or not to claim your Publons credit. I understand this statement.

Yes Choose an item.
