# Supplementary material for: RepeatFiller newly identifies megabases of aligning repetitive sequences and improves annotations of conserved non-exonic elements
Source: Gigascience. 2019 Nov 19;8(11):giz132. doi: 10.1093/gigascience/giz132 (PMC6862929; doi:10.1093/gigascience/giz132)
Supplement: giz132_Reviewer_2_Report_Original_Submission — Ge Tan, Ph.D. -- 8/20/2019 Reviewed [file giz132_reviewer_2_report_original_submission.pdf]

Reviewer Report

Title: RepeatFiller newly identifies megabases of aligning repetitive sequences and improves annotations of conserved non-exonic elements

Version: Original Submission **Date:** 8/20/2019

Reviewer name: Ge Tan, Ph.D.

Reviewer Comments to Author:

The authors presented a neat way of rescuing the potentially missing whole genome alignments for the repeat masked regions. I have a few questions and concerns regarding this manuscript.
1. The authors demonstrated the application of RepeatFiller on human hg38 against other 20 mammals. I am curious to see how well RepeatFiller can be applied to other vertebrates or even invertebrates? How it copes with highly fragmented assembly? The guidelines of how to choose the options on other cases are highly desired from the authors.
2. In "Generating pairwise genome alignments", the authors used the same lastz alignment parameters and default scoring matrix for genome alignment against hg38. I wonder why this is case? For instance, in UCSC, Human vs. Rhesus uses the human_chimp.v2.q scoring matrix for closer species. I expect to get many spurious alignments from Human vs. Rhesus alignment, hence much lower "added aligning sequence" for Rhesus in Figure 2.
3. Can authors explain a bit on what factors might be related to amount of "added aligning sequence" in Figure 2? I would expect to see a higher recovery rate for the species that are more evolutionary distant and have better assembly quality, because the co-linear alignments should be anchored better in the first round of alignment. However, it doesn't seem to be the case.
4. In terms of the novel repeat-derived conserved non-exonic elements, further details of those CNEs is needed. I have concerns about how genuine those CNEs are. Do they similar characteristics, compared to other normal CNEs? Are these repeat-derived CNEs also AT-rich? Do the widths of CNEs follow a power-law distribution? Is there any locus where only repeat-derived CNEs exist?

Level of Interest

Please indicate how interesting you found the manuscript: Choose an item.

Quality of Written English

Please indicate the quality of language in the manuscript: Choose an item.

Declaration of Competing Interests

Please complete a declaration of competing interests, considering the following questions:

- Have you in the past five years received reimbursements, fees, funding, or salary from an organisation that may in any way gain or lose financially from the publication of this manuscript, either now or in the future?
- Do you hold any stocks or shares in an organisation that may in any way gain or lose financially from the publication of this manuscript, either now or in the future?
- Do you hold or are you currently applying for any patents relating to the content of the manuscript?
- Have you received reimbursements, fees, funding, or salary from an organization that holds or has applied for patents relating to the content of the manuscript?
- Do you have any other financial competing interests?
- Do you have any non-financial competing interests in relation to this paper?

If you can answer no to all of the above, write 'I declare that I have no competing interests' below. If your reply is yes to any, please give details below.

I declare that I have no competing interests

I agree to the open peer review policy of the journal. I understand that my name will be included on my report to the authors and, if the manuscript is accepted for publication, my named report including any attachments I upload will be posted on the website along with the authors' responses. I agree for my report to be made available under an Open Access Creative Commons CC-BY license (http://creativecommons.org/licenses/by/4.0/). I understand that any comments which I do not wish to be included in my named report can be included as confidential comments to the editors, which will not be published.

Choose an item.

To further support our reviewers, we have joined with Publons, where you can gain additional credit to further highlight your hard work (see: https://publons.com/journal/530/gigascience). On publication of this paper, your review will be automatically added to Publons, you can then choose whether or not to claim your Publons credit. I understand this statement.

Yes Choose an item.
