# Supplementary material for: RepeatFiller newly identifies megabases of aligning repetitive sequences and improves annotations of conserved non-exonic elements
Source: Gigascience. 2019 Nov 19;8(11):giz132. doi: 10.1093/gigascience/giz132 (PMC6862929; doi:10.1093/gigascience/giz132)
Supplement: giz132_Supplemental_Files [file PointByPointResponse.pdf › PointByPointResponse.pdf]

# Point by point response

**Editorial request:**
Please register any new software application in the SciCrunch.org database to receive a RRID (Research Resource Identification Initiative ID) number, and include this in your manuscript. Please also register it with the ELIXIR run bio.tools registry and quote the number too. This will facilitate tracking, reproducibility and re-use of your tool.

We have registered RepeatFiller with both databases:
SciCrunch.org ID is SCR_017414 and ELIXIR bio.tools registry ID is biotools:RepeatFiller and added this information to the Data Availability section.

# Reviewer reports:

**Reviewer #1:**
"RepeatFiller newly identifies megabases of aligning repetitive sequences and improves annotations of conserved non-exonic elements" is a nice demonstration of a general software engineering approach to addressing high complexity problems, it reduces the complexity by making sensible cuts in the input data to make the problem feasible. In the case of RepeatFiller, sensible cuts are placed at the boundaries of otherwise uniquely unalignable segments of genomes based on anchor regions that are uniquely unalignable. This approach reduces the seed complexity of alignment from genome scale down to a scale that can be properly considered by standard aligners.

The results of using this approach show that known interspecies-conserved regions are both missed without the use of this technique due to aligners ignoring high-repeat density regions for alignment seeding, but also conversely that known conserved regions have their level of conversation overestimated due to absence of more divergent alignments when using traditional alignment methods. Given the apparent importance of repetitive elements in the evolution of eukaryotic gene regulation the tool described here is likely to find interesting new regulation systems and refine understanding of existing systems.

We thank the reviewer for these kind words.

The manuscript is well written with only a minor grammatical error: "Requiring minimum size of 30 bp, application [of] RepeatFiller led to the identification of ...".

Thank you for noticing that. We have fixed that mistake.

It would be helpful for the authors to provide better instructions for building the dependencies (downstream dependencies of Kent's source are not listed either on the project page or in the build instructions for the Kent source). It should be noted that attempting to build Kent's source fails for absence of dependencies that appear not to be used by RepeatFiller (MySQL). The provided pre-compiled binaries do not run, again due to a missing (or mis-versioned)

dependency (libssl). Because of these issues it may be worth vendoring a cut down Kent source with the RepeatFiller source.

Yes, the kent source code package requires many dependencies, which makes it hard to install. As suggested, we have therefore changed makefiles (and *.mk files) and removed lots of directories in the code base and generated a light-weight kent source package that does not need the MySQL library anymore. We were not able to remove the dependency on libssl, which appears to be an integral part of the code, as this would involve re-writing parts of the code. However, libssl is either already included in many Linux installations or otherwise easy to install. We have updated everything on github.

**Reviewer #2:**
The authors presented a neat way of rescuing the potentially missing whole genome alignments for the repeat masked regions. I have a few questions and concerns regarding this manuscript.

1. The authors demonstrated the application of RepeatFiller on human hg38 against other 20 mammals. I am curious to see how well RepeatFiller can be applied to other vertebrates or even invertebrates? How it copes with highly fragmented assembly? The guidelines of how to choose the options on other cases are highly desired from the authors.

We agree with the reviewer that it is important to test RepeatFiller on a wider range of species exhibiting different genome characteristics. As suggested, we have now applied RepeatFiller to three fragmented mammalian genomes (Parnell's bat, rock hyrax and kangaroo rat with scaffold N50 values of <36 kb).
In addition, we computed alignments between other vertebrate and invertebrate genomes and applied RepeatFiller to birds (zebra finch to chicken), reptiles (green anole lizard to bearded dragon; American alligator to painted turtle) and invertebrates (Drosophila pseudoobscura to D. melanogaster).
The results are shown in a new Figure 2 panel B, in a new Figure 3 and in a new Supplementary Table 2.

First, we found that assembly contiguity is a factor that influences the amount of newly-added alignments for mammals, and therefore we have integrated this analysis in the new paragraph that investigates which factors influence differences in the amount of added alignments (please see our response to point 3 below). Nevertheless, we showed that RepeatFiller still adds >32 Mb of new alignments for the fragmented mammalian assemblies. Based on these new results, we conclude that RepeatFiller can find new alignments "for both fragmented or contiguous mammalian genomes" (conclusion sentence on page 5).

Second, RepeatFiller also detected >1 Mb of new alignments for the bird and reptile comparisons and 230 kb for the Drosophilid comparison. As we write in a new section, this is partially related to how repeat-rich the genomes are, but repeat content is not the only factor. Nevertheless, RepeatFiller added >1 Mb in previously-undetected alignments for birds and reptiles, which indicates that application of RepeatFiller to non-mammalian genomes can also improve the completeness of genome alignment with little extra runtime.

"**RepeatFiller also detects additional alignments for non-mammalian genomes**
The majority of the newly-detected alignments between mammalian genomes overlap transposable elements or other repeats. One would therefore expect that RepeatFiller application to alignments of species with less repeat-rich genomes detects fewer novel alignments. To test whether this is generally true, we applied RepeatFiller to alignments of birds (zebra finch aligned to chicken), reptiles (green anole aligned to bearded dragon; American alligator aligned to painted turtle), and insects (Drosophila pseudoobscura aligned to D. melanogaster). For birds and insects, whose genomes generally consist of <20% repeats [27-29], RepeatFiller added few new alignments (1.9 Mb for birds representing 0.18% of the chicken genome, 231 kb for Drosophilids representing 0.16%

of the D. melanogaster genome) (Figure 3, Supplementary Table 2). For reptiles, RepeatFiller added 4.5 Mb of new alignments to the green anole - bearded dragon genome alignment (0.26% of the bearded dragon genome) and 14.5 Mb to the alligator - turtle alignment (0.61% of the turtle genome) (Figure 3, Supplementary Table 2). Thus, despite the fact that reptile and mammal genomes generally have a similar repeat content of ~30-50% [28, 30], RepeatFiller added fewer alignment for reptiles compared with mammals. This shows that other factors in addition to genomic repeat content also influence the amount of added alignments. Nevertheless, more than one megabase of previously-undetected alignments for birds or reptiles show that RepeatFiller, with little additional runtime, can also improve the completeness of aligning repetitive regions between species in these groups.
”

2. In "Generating pairwise genome alignments", the authors used the same lastz alignment parameters and default scoring matrix for genome alignment against hg38. I wonder why this is case? For instance, in UCSC, Human vs. Rhesus uses the human_chimp.v2.q scoring matrix for closer species. I expect to get many spurious alignments from Human vs. Rhesus alignment, hence much lower "added aligning sequence" for Rhesus in Figure 2.

Compared to the UCSC parameters, the parameters we have used to align rhesus (and other mammals) to human are generally more sensitive. Specifically, apart from using the lastz default scoring matrix, which penalizes substitutions less than the human_chimp.v2.q scoring matrix, we also used score thresholds for ungapped/gapped local lastz alignments of 2400/3000, while UCSC used score thresholds of 4500/3000.

To test how much the results differ when using the UCSC parameters and the human_chimp.v2.q scoring matrix, we re-ran the human-rhesus alignment with UCSC parameters and tested RepeatFiller. As expected, before applying RepeatFiller, our more sensitive parameters detect more alignments, as an additional 45 Mb of the human genome aligned to rhesus (2681 vs. 2635 Mb of the human genome aligned to rhesus with our vs. UCSC parameters).

Importantly, as shown in Supplementary Table 1, RepeatFiller added >20 Mb to both sets of chains. From this, we concluded that applying RepeatFiller is also useful for chains generated with UCSC parameters. This analysis has been added to Supplementary Table 1 and to the text on page 9:
“We also tested aligning human and rhesus using K = 4500, L = 3000, Y = 15000, H = 2000 and the UCSC human_chimp.v2.q scoring matrix and found that applying RepeatFiller to these chains also added a similar amount (25.2 vs. 22.4 Mb) of newly aligning sequence (Supplementary Table 1).”

| | | | | | Aligning bases newly identified by RepeatFiller all bases | |
|---|---|---|---|---|---|---|
| commmon species name | assembly (UCSC) | RepeatFiller run time (CPU hours) | Human bases that align to another species WITHOUT RepeatFiller | Human bases that align to another species WITH RepeatFiller | bp | % of human genome |
| **Difference between our and UCSC alignment parameters** | | | | | | |
| Rhesus, our parameters (K = 2400, L = 3000, Y = 9400, H = 2000, default matrix) | rheMac8 | | 2,680,545,226 | 2,702,943,250 | 22,398,024 | 0.70 |
| Rhesus, UCSC parameters (K = 4500, L = 3000, Y = 15000, H = 2000, human_chimp.v2.q matrix) | rheMac8 | | 2,635,190,553 | 2,660,384,934 | 25,194,381 | 0.79 |
| | | **Difference** | 45,354,673 | 42,558,316 | - 2,796,357 | |

3. Can authors explain a bit on what factors might be related to amount of "added aligning sequence" in Figure 2? I would expect to see a higher recovery rate for the species that are more evolutionary distant and have better assembly quality, because the co-linear alignments should be anchored better in the first round of alignment. However, it doesn't seem to be the case.

Thank you for this interesting question. We analyzed whether assembly contiguity (using contig and scaffold N50 values), the percent of the query genome that is repeat-masked, and the evolutionary distance (branch length between human and the species) influence the amount of newly-added alignments. It should be noted that we excluded the rhesus macaque in these analysis as this species represents an outlier, likely because it is very closely-related to human and both genomes are highly similar. Indeed, considering all 20 mammals, we obtain a correlation of 0.46 between the amount of added alignments and the evolutionary distance; however, this is entirely driven by rhesus, as excluding rhesus results in a non-significant negative correlation of -0.14.

We describe the influence of assembly contiguity and percent of the genome that is repeat-masked in a new text paragraph on page 5 and Supplementary Table 1:
"Next, we investigated what factors are associated with differences in the amount of newly-aligned sequences per species. In these tests, we excluded rhesus macaque that is closely related to human as an outlier. First, as expected, the percent of the genome that is repeat-masked significantly influences the number of newly-aligned bases (P=0.0004, Supplementary Table 1), which supports our assumption that the initial alignment step misses alignments due to repeat-masking rather than sequence dissimilarity. Second, we investigated how assembly contiguity of the query genome influences the results. We found that the scaffold N50 value has a small but non-significant effect on the amount of added aligned bases (P=0.067, Supplementary Table 1). Since chains cannot span scaffold boundaries, we further tested the influence of scaffold N50 values by applying RepeatFiller to alignments of three fragmented mammalian assemblies: Parnell's mustached bat, rock hyrax and kangaroo rat, which have scaffold N50 values between 23 and 36 kb. While RepeatFiller still added a substantial amount of new alignments, ranging from 32 Mb to 35 Mb (Figure 2B, Supplementary Table 2), more new alignments were generally found for more contiguous mammalian assemblies."

4. In terms of the novel repeat-derived conserved non-exonic elements, further details of those CNEs is needed. I have concerns about how genuine those CNEs are. Do they similar characteristics, compared to other normal CNEs? Are these repeat-derived CNEs also AT-rich? Do the widths of CNEs follow a power-law distribution? Is there any locus where only repeat-derived CNEs exist?

First, consistent with previous findings that CNEs are in general more AT-rich, we found that the newly-detected CNEs are more AT-rich than randomly selected genomic regions.

The same is true for CNEs already detected in the multiple alignment without applying RepeatFiller.

Second, we found that the newly-detected CNEs are shorter (median 41 vs. 50). Nevertheless, 1515 newly-detected CNEs are at least 100 bp long with a maximum of 760 bp. The CNE widths also follow a power law distribution.

These two points have been added as a new Supplementary Figure 1 and to the text on page 6:
"With a median size of 41 bp, these novel CNEs are shorter than CNEs already detected in the non-RepeatFiller alignments (median 50 bp, two-sided Wilcoxon rank sum test P<$e^{-16}$, Supplementary Figure 1), likely because most of the longer conserved regions were already in the initial genome-wide alignment step. Consistent with previous findings that CNEs are in general more AT-rich [36], we found that the novel CNEs are more AT-rich than randomly selected, non-conserved genomic regions (two-sided Wilcoxon rank sum test P<$e^{-16}$, Supplementary Figure 1)."

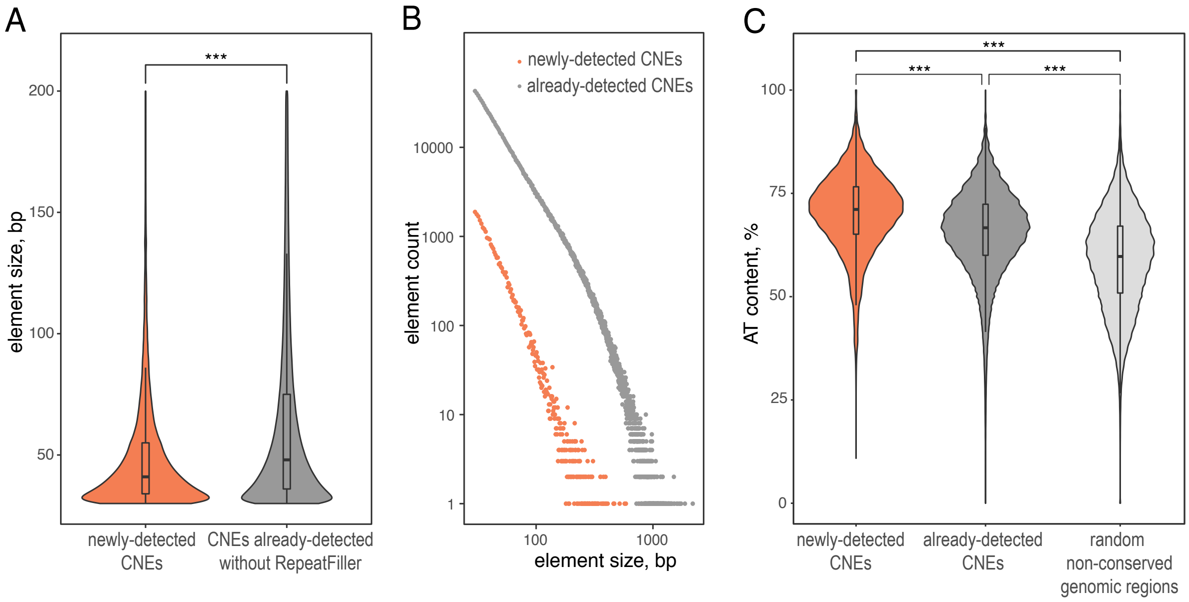


**Supplementary Figure 1:** Properties of newly-detected CNEs.
We compared CNEs already-detected in the non-RepeatFiller alignments (dark grey) to 30167 novel CNEs that were only detected after applying RepeatFiller (orange).
(A) Violin plots overlaid by box plots show that newly-detected CNEs are significantly shorter (median 41 vs. 50 bp, average 50.3 vs. 76.7 bp) and lack very large CNEs (maximum 760 vs. 2193 bp). For visualization, the shown distributions are cut at 200 bp.
(B) The size distribution of newly- and already-detected CNEs is similar to a power law distribution.
(C) Violin plots overlaid by box plots show the percent A+T bases per CNE. Newly- and already-detected CNEs are more AT-rich than randomly selected, non-conserved genomic regions that have the same size as the already-detected CNEs. We repeated

the sampling of non-conserved genomic regions 10 times and found a highly significant difference in each case.
***: $P<e^{-16}$ in a two-sided Wilcoxon rank sum test.

Finally, we investigated whether there are larger regions that only contain newly-detected CNEs. The largest region is a ~800 kb region on chr19:53052185-53849181; however, this one only contains 3 novel CNEs. We therefore filtered for regions with 10 or more novel CNEs and sorted by size. The top 5 regions are listed below. We inspected these regions and all are outside of genes, but apart from that there is no obvious feature that stands out. Given the small size of these regions, we think it is expected by chance to detect smaller regions that only contain newly-detected CNEs.

| | | |
|---|---|---|
| 22886 bp: chrX | 24086045 | 24108931 (11 CNEs) |
| 17881 bp: chrX | 49006943 | 49024824 (11 CNEs) |
| 16504 bp: chr17 | 38628775 | 38645279 (11 CNEs) |
| 14257 bp: chr1 | 38396059 | 38410316 (10 CNEs) |
| 14025 bp: chr6 | 98147310 | 98161335 (12 CNEs) |
